# High-Resolution Images with Minimum Energy Dissipation and Maximum Field-of-View in Camera-Based Wireless Multimedia Sensor Networks

**DOI:** 10.3390/s90806385

**Published:** 2009-08-19

**Authors:** Hadi S. Aghdasi, Pouya Bisadi, Mohsen Ebrahimi Moghaddam, Maghsoud Abbaspour

**Affiliations:** Faculty of Electrical and Computer Engineering, Shahid Beheshti University, G.C., Tehran, Iran; E-Mails: aghdasi@sbu.ac.ir (H.S.A.); po.bisadi@mail.sbu.ac.ir (P.B.); m_moghadam@sbu.ac.ir (M.E.M.)

**Keywords:** camera-based sensor networks, high-resolution and wide images, two-tier network topology, image stitching, camera selection problem, minimum wasted energy

## Abstract

High-resolution images with wide field of view are important in realizing many applications of wireless multimedia sensor networks. Previous works that generally use multi-tier topology and provide such images by increasing the capabilities of camera sensor nodes lead to an increase in network cost. On the other hand, the resulting energy consumption is a considerable issue that has not been seriously considered in previous works. In this paper, high-resolution images with wide field of view are generated without increasing the total cost of network and with minimum energy dissipation. This is achieved by using image stitching in WMSNs, designing a two-tier network topology with new structure, and proposing a camera selection algorithm. In the proposed two-tier structure, low cost camera sensor nodes are used only in the lower-tier and sensor nodes without camera are considered in the upper-tier which decreases total network cost as much as possible. Also, since a simplified image stitching method is implemented and a new algorithm for selecting active nodes is utilized, energy dissipation in the network is decreased by applying the proposed methods. The results of simulations supported the preceding statements.

## 1. Introduction

Wireless multimedia sensor networks (WMSNs) are a new and emerging type of sensor networks that contain sensor nodes with capability of obtaining multimedia content such as video and audio streams, still images, and scalar sensor data from the environment [1,2]. Recently, some new applications for wireless multimedia sensor networks such as multimedia monitoring systems [3], advanced health care delivery [4], automated assistance for the elderly and family monitors [5], traffic avoidance, enforcement, and control systems [6] have appeared.

Capturing images from a desired scene is necessary in many monitoring applications. By increasing the field of view and resolution of these images, more applications are realizable. One common way to achieve this goal is to use strong and costly cameras in a single-tier network topology that leads to more energy usage and cost. According to the characteristics of WMSNs, utilizing only these cameras is not acceptable. To have more feasible networks, most applications use different type of camera sensor nodes that are organized in multi-tier topologies. However, available multi-tier topologies have more than two tiers with high cost camera sensors in upper tiers which increases the network cost and complicates the network management.

In this paper, a methodology to provide high resolution images with wide field of view in Camera-Based WMSNs is presented that have following contributions: (i) utilizing an image stitching method in WMSNs, (ii) designing a two-tier network topology with new structures which has camera sensor nodes only in lower-tier, (iii) presenting a method that let upper-tier sensor nodes perform a simplified image stitching, and (iv) presenting an innovative algorithm for lower-tier camera nodes to have a good coverage of field for longer time by selecting them fairly.

The presented network topology in this paper consists of just two tiers. Unlike common multi-tier WMSN topologies this topology has camera sensor nodes only in its lower-tier. Also, in the other topologies, multiple tiers are equipped with cameras and the capabilities of camera sensor nodes in them increase from lower-tier to Sink. As lower-tier camera nodes are low cost, it is possible to have high density in this tier and consequently highly overlapped coverage. This property which decreases the coverage failure is utilized to provide a suitable coverage for a longer time. The presented network upper-tier includes stronger sensor nodes without camera. This two-tier topology and its intra-tier and inter-tier communication methods have been described in Section 3.

Having numerous low-resolution images, provided by many low cost sensor nodes in lower-tier, is not acceptable for many monitoring applications. Therefore, to create a high resolution and wide image, this paper simplifies and utilizes an image stitching method in a specific way which has been adapted to WMSNs constraints. As image stitching procedure requires a lot of processing overhead, the stitching method is distributed between Sink and upper-tier sensor nodes such that Sink performs main steps of methodology like image processing and some specific extracted information is sent to related nodes in the upper-tier by Sink. Having this information, upper-tier sensor nodes are able to perform stitching procedure with low computational overhead in the next times. By stitching images which have overlaps, the size of whole data that must be transmitted hierarchically through upper-tier to Sink decreases. Consequently, as the main reason of energy consumption is data transmission, decreasing the data transmission size prolongs the lifetime of upper-tier. More details about the stitching method are explained in subsection 4.1.

Energy consumption is the most important issue in WMSNs. Because the density of camera sensor nodes in the lower-tier is high and areas covered by them have large overlaps, therefore, selecting an appropriate subset of them that provides an acceptable coverage of desired area must be considered. Selecting a proper subset of nodes firstly satisfies the Sink request for the coverage of a desired area, and secondly, it puts unselected nodes in sleep mode and saves their energy, because not setting all nodes in active mode will prolong the lifetime of the lower-tier. In subsection 4.2, an algorithm named Fair Camera Node Selection algorithm (FCNS) performed by upper-tier sensors is presented to provide a good coverage of field in a way that each node is selected an almost equal number of times in multiple requests from Sink. Uniform selection of camera sensor nodes that avoids the early failure of some of them makes good coverage available for a longer time. A comprehensive description of its construction method is presented in subsection 4.2.1.

All in all, by using image stitching in WMSNs based on the contributions presented in this paper, high-resolution images with wide field of view are provided while WMSNs constraints are considered. Moreover, contributed methods reduce energy dissipation and let the network preserves its functionality for a longer period of time. However, presented methods have high performance when there are not a lot of changes in the network topology like camera sensors locations. Therefore, these methods fit WMSNs applications whose sensor nodes have no mobility and whose cameras are placed in a fixed location. Analysis and simulations yielded expected results.

The rest of the paper is organized as follows. Section 2 presents the related work. The topology of Two-Tier Camera-Based WMSNs is presented in Section 3. The methodology of generating high-resolution images with minimum energy waste in camera-based WMSNs, including image stitching technique and camera sensor node selection algorithm, is introduced in Sections 4. Section 5 provides simulation and experimental results. Finally, Section 6 concludes the paper.

## 2. Related Work

Recently, maximizing the coverage of an area, improving the resolution and increasing the field of view in WMSNs have all been studied in great depth. Equipping sensor nodes used in regular WMSNs with some extra resources or using image processing methods are instances of methods used in previous works which tried to satisfy these goals. Cameras which have the ability of pan, tilt, and zoom as well as moveable platforms are samples of resources that sensor nodes are equipped with [7–9]. Creating accurate or high-resolution images by processing a sequence of rough images, utilizing image fusion, and using new compression methods over row data are instances of image processing ways [10,11]. Some of related literature is described in the following paragraphs.

In [7], the authors have maximized multimedia coverage in WMSNs by considering video sensor orientation. This method finds the best direction of camera sensors dynamically and depends on the circumstances not on setting camera sensors orientation once they have been deployed. The distributed algorithm which is proposed in order to perform this goal has two main steps: (i) minimizing the effects of occlusion in the environment and (ii) improving the cumulative quality of the information sensed from the region of interest. This algorithm improves robustness of WMSNs, because the direction of camera sensors could be updated after nodes fail due to the battery outage or external effects.

In [8], the authors have improved their previous work in [7] such that each sensor node determines the most beneficial orientation for its multimedia sensor so that the entire image of a field can be constructed using low-resolution snapshots from multiple sensors. In overall, this approach has some benefits: (i) the proposed algorithm is fully distributed using local information, so communication overhead is incurred only among neighboring nodes, (ii) with the flexibility to adjust orientations of the multimedia sensors, multimedia sensor nodes update the orientation of multimedia sensors on the fly to increase the multimedia coverage significantly, (iii) overlapped and occluded regions in the sensing field can be decreased by collecting the current pose of neighboring nodes and (iv) coverage is increased even for sparse networks by using self-orientation instead of random orientations when arbitrary obstacles exist in the sensor field.

Although the mentioned approaches have solved some problems in WMSNs, they have some weak points. First and foremost, these approaches are based on single-tier topology, whereas according to [12] the topology of WMSNs must be multi-tier in order to achieve a balance between cost, coverage, functionality, and reliability. Also, these approaches are costly because they use strong nodes which are also equipped with extra resources like a rotatable platform for cameras. On the other hand, they are not suitable for real-time application because changing the orientation of camera sensors is performed by mechanical devices. Moreover, since there is no guarantee of covering the area related to a node by its neighbors, these approaches tried to reduce the number of sensor nodes, so the reliability of the WMSN is decreased. Therefore, if a node fails, some part of the field will not be covered.

In [9], authors discussed a system architecture that uses controlled motion to provide virtual high-resolution in a network of camera sensors. The motion abilities have been added to camera sensors include pan, tilt, and zoom, help avoiding obstacles and camera overlap. After detecting an interesting phenomenon, camera sensor tries to provide a new image of it which has acceptable level of details for recognition. This method is not suitable for real-time application, because if the tolerable delay in sensing is small, then only a limited range of motion may be feasible and capturing an image with adequate details for recognition is impossible. On the other hand, due to the existence of motion abilities in the sensors, this approach requires more energy consumption.

An application-aware routing algorithm has been presented in [13] which decreases camera sensor overlap and optimizes the solution of the coverage problem in video-based sensor networks. In this method, it is assumed that all camera nodes are located on a plane surface like the ceiling of the monitored room and capture the images from a parallel plane. Since the topology of presented approach in [13] is single-tier, many sensor nodes get involved in data transmission and consequently the lifetime of the network decreases, although a compatible routing algorithm for WMSNs is presented. In addition, in spite of selecting suitable subset of nodes by routing algorithm, all raw data transit through the network because camera sensor nodes have low processing capabilities and cannot filter undesired data before sending them.

Recently in [14] an image registration method for low-resolution visual sensor networks has been presented which is based on registering two images. However, the topology of sensor networks and communication criteria from image capturing node to Sink is not addressed in this work. But, as far as we know, optimization of energy consumption and achieving best coverage and resolution depend highly on network topology, communication methods between sensor nodes, and camera sensor selection algorithms and techniques. In addition, the affine transform used in image registration is not suitable for sensor networks because camera nodes directions are slightly different. Finally, in order to create a large view of the area under observation in many applications, more than two images are needed to be registered.

## 3. Two-Tier Camera-Based WMSNs Topology

Considering the mentioned applications for Camera-Based WMSNs, monitoring is the common aspect of the most of these applications. High coverage, high reliability, low cost, and high functionality are essential for achieving higher performance in monitoring applications [15].

According to the literature, almost all of the single-tier sensor networks satisfy merely one of the mentioned essential items that are necessary for improving performance in monitoring applications. For example, using many low cost cameras as sensor nodes increases the coverage and decreases the functionality because of limited capabilities of these nodes. On the other hand, using more powerful and expensive nodes increases the functionality but achieving the reliability by increasing the network density would be really costly.

Multi-tier topologies have succeeded in creating a balance between cost, functionality, reliability, and coverage. For example, the lowest tier contains cheap sensors with poor functionality and quality while the higher one compensates for the lack of these items by stronger and more expensive nodes. Some practical examples of Multi-tier WMSNs are low-resolution-camera-based sensor nodes such as low-power Motes [16] nodes that are equipped with Cyclops cameras [17] in the lowest tier, stronger camera-based sensor nodes like Imote2 [18] nodes equipped with CMUCam3 [19], Mesheye [20], and Panoptes [21] which are placed in the higher tier and finally the nearest tier to Sink includes strongest camera-based sensor nodes like Stargate-XScale nodes [22] with Web-Cam. Also, in [23] an experimental multi-tier architecture has been introduced which is called SensEye.

All in all, the purpose of multi-tier topologies is providing a balance between cost, functionality, coverage, and reliability to realize many WMSNs applications. Therefore, the presented topology is a multi-tier topology which unlike typical WMSNs topologies has only two tiers. The main differences between this topology and the others are that it has just low cost camera sensor nodes in lower-tier and its upper-tier consists of stronger nodes without camera that perform tasks like simplified image stitching and lower-tier management. Obviously, presented two-tier topology which has no camera in upper-tier cannot acquire a high-resolution image without using Image Stitching. The structure of upper-tier, lower-tier and the communication methods are explained in subsections 3.1 and 3.2 respectively.

### 3.1. Proposed Upper-Tier Structure: Wireless Network of Stronger Sensor Nodes without Camera

Sensor nodes in the proposed upper-tier are not equipped with cameras but they have stronger processors, communication units, and more storage capacity. Stargate [22], Imote2 [18], and Yale XYZ [24] are practical instances of stronger sensor nodes. Their task is lower-tier management, information gathering and processing such as simplified image stitching, and transmitting the final result of this process to Sink. Moreover, all nodes in this tier are equipped with Global Positioning System (GPS). Accordingly, Sink knows their geographical position and easily sends its queries to the related cluster head.

Each of the stronger sensor nodes has the responsibility of multiple camera nodes in the lower-tier. Therefore, upper-tier contains fewer sensor nodes and less density compared to the lower-tier. At the beginning, stronger sensors nodes are placed by network manager so as their density would be uniform in the total covering field. However, the radio-covered areas by upper-tier sensor nodes have overlaps to increase the tolerance of the whole network versus a failure of upper-tier nodes, to prolong network lifetime an appropriate subset of nodes in this tier must be chosen to be in active mode. Clustering algorithms are suitable for selecting this subset. After determining the number of clusters, which depends on the application, and performing clustering action, it is adequate to set only one node (cluster head) in each cluster to active mode and leave the others in sleep mode. Moreover, to achieve good coverage; firstly, all nodes in the upper-tier should be classified into clusters with almost equal number of members; secondly, the selection of cluster heads (active nodes) should be in a way that their locations in upper-tier become uniform. The proposed algorithms in [25–27] are instances that can be employed here.

**Figure 1.**
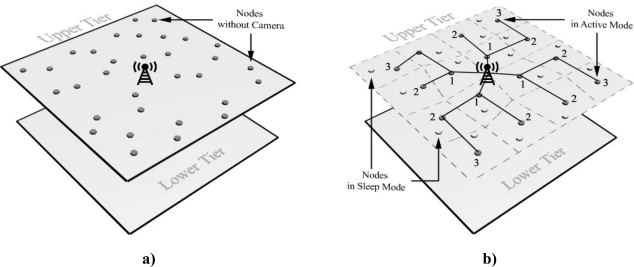
a) Symbolic figure of proposed upper-tier structure at the beginning. b) Symbolic figure of proposed upper-tier structure after clustering and routing.

After clustering and selecting cluster heads, a routing protocol is necessary for data transmission between each cluster head and Sink. As we know, hierarchical protocols are suitable for this tier because of their speed and low energy consumption. The protocols in [28,29] are instances which are suitable for this purpose. Figure 1 depicts the structure of the upper-tier.

### 3.2. Proposed Lower-Tier Structure: Wireless Network of Sensor Nodes Equipped with Cameras

Lower-tier is constructed of sensor nodes that are in contact with the physical environment. Therefore, in order to achieve practical multimedia applications in WMSNs, sensor nodes in this tier are equipped with cameras. Cyclops low-power camera sensor [17], CMUCam3 [19], Mesh-Eye [20], and Panoptes [21] are practical instances that can be utilized as camera nodes in this tier.

**Figure 2.**
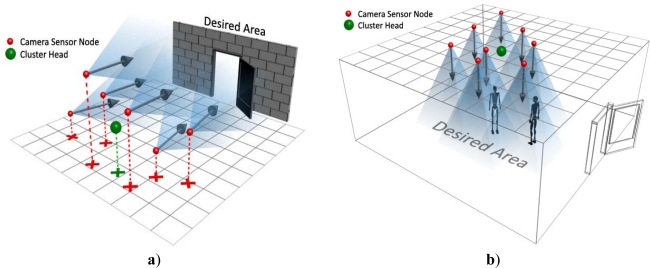
a) Outdoor placement of a cluster. b) Indoor placement of a cluster.

The task of each camera node is to capture still images and transmit them to its cluster head in the upper-tier. The connection between lower-tier and upper-tier is single hop and there is no interactivity between camera nodes. In order to have maximum coverage of environment, the number of camera nodes in this tier is high; therefore, the fault tolerance increases because if one of camera sensors fails, each part of area will be covered by another camera. Also at first, all camera sensors are placed by network manager in a way that their density would be uniform and the field is covered completely. In addition, the adjacent cameras are placed in almost same directions; it means that images of camera sensors which are nearer to each other have more overlaps and can be stitched to each other. Figure 2 a,b shows the placement of indoor and outdoor applications. As it is depicted in these figures, in some applications sensor are placed in a flat area while they have a 3-D placement in some others.

**Figure 3.**
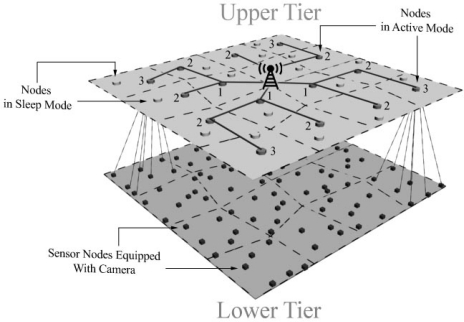
Symbolic figure of proposed two-tier camera-based WMSN topology.

Each camera node covers a small section of environment, while the application purpose is to get high-resolution images that cover large areas. Hence, the nodes of this tier must be clustered based on their location to let the cluster head perform this task properly by stitching the images of each cluster. The acquired high-resolution image of each cluster head is independent from the other one and Sink requests it by sending a query to the corresponding cluster head. Supposing that nodes are placed with uniform density in this tier, if the number of node members in each cluster is equal, the areas which are covered by each cluster will be roughly the same size. As mentioned in subsection 3.1, the location of cluster heads in upper-tier are uniform; so, if every camera node which can connect to more than one cluster head selects the cluster head with fewer members, then the lower-tier clusters will automatically have almost equal member counts. To realize the mentioned goal, one possible algorithm used in this paper is Minimal Cardinality Variance Clustering (MSVC) [30]. In this algorithm, camera nodes are aware of their options for connecting to upper-tier cluster heads. Each camera node opts to connect to the cluster head which has the least number of child nodes. Obviously, if there is only one choice for connection, the camera node will connect to that cluster head. With regards to the structure of upper-tier and lower-tier, the overall view of two-tier heterogeneous sensor networks topology and its inter-tier or intra-tier communication is depicted in Figure 3.

## 4. High-Resolution Images in Camera-Based WMSNs

The resolution and field of view of camera sensor nodes in WMSNs are low, while most applications of WMSNs need a high field of view with high quality. According to the literature, the previous methods solved this problem by using a hierarchy of cameras which contains stronger and more expensive cameras in higher tiers of it. But, the presented method uses image stitching to produce high quality images which cover wide areas by utilizing low cost camera sensor nodes just in lowest tier. The image stitching is the process of combining multiple images in order to produce an image with higher resolution and wider field of view.

Due to camera sensor nodes limitations and the complexity of image stitching process, in order to reduce the volume of data transmission and prolonging network lifetime, process of creating high-resolution images with maximum field of view consists of these stages. By the first request from Sink to the desired cluster head, the cluster head gathers the low-resolution images of all camera nodes in its cluster and sends all of them to Sink. Sink stitches these images to produce a high-resolution image as the result of the first query. A lot of efforts in image stitching process are taken to find transformation matrices. Hence, having these matrices beforehand makes stitching process really less complicated. So, Sink calculates these matrices and sends them to the cluster head to perform stitching in the subsequent queries (Details are explained in subsection 4.1). Also, based on stitched image a Coverage Data Structure is created by Sink (Details are explained in subsection 4.2.1). By the next queries, using this Coverage Data Structure and running Fair Camera Node Selection algorithm, the cluster head selects just a subset of camera nodes and gathers their images (subsection 4.2.2). Because the cluster head has transformation matrices, a simple procedure is adequate to stitch images of this selected subset before sending them to Sink.

### 4.1. Image Stitching Technique

As Sink has no resource limitation, it performs image stitching process on the low-resolution images of a desired cluster head initially, extracts some necessary information and sends them to that cluster head. After receiving this information by the cluster head, it would be capable of stitching images by a simple process. Considering the nature of WMSNs, it is necessary for their topology to be reconfigured at periods which depend on the network application. The information transmitted by Sink is valid as long as the network topology has not been changed. On the other hand, after reconfiguration of the network topology, if it is the first time that Sink sends a query to a cluster head, it should stitch the received low-resolution images to send necessary information to the cluster head. Now, having this information, the cluster head stitches images with low computational overhead. Image stitching process that is used in extracting this information is explained as follows.

Almost all image stitching methods can be put in one of the two broad categories: Direct Methods [31–34] and Feature-based methods [35–38]. Direct methods shift or rotate images related to each other and search for the best pixel-to-pixel match by minimizing a predefined error metric. But, Feature-Based methods extract features of each image at first and use them to register images in the next steps. So, these methods are faster whereas they suffer from low accuracy. However, after the presentation of SIFT feature extraction method [39], the accuracy problem has been solved. Recently, SIFT is utilized in many applications, especially in image stitching. Therefore, today, Feature-based methods are very popular in image stitching techniques.

Nearly all image stitching methods need an initialization which must be typically done by the user such as approximately aligning the images or fixing image ordering. For example, REALVIZ Stitcher version 4 [40] has a user interface to roughly position the images with a mouse before automatic registration proceeds. Also, the PhotoStitch software bundled with Canon digital cameras requires a horizontal or vertical sweep, or a square matrix of images [38]. But the method presented by Matthew Brown and David G. Lowe [37,38] is a fully automated panoramic image stitching which does not need any human input. This method is insensitive to the ordering, orientation, scale, and illumination of the input images.

Consequently, according to the camera-based WMSNs characteristics including: variant scale, orientation and illumination of camera sensor images and the fact that WMSNs are autonomous systems, Brown and Lowe’s method is suitable for image stitching in these types of networks. However, in this paper a simplified Brown and Lowe’s stitching method is proposed to satisfy camera-based WMSNs limitations. Feature matching, Image matching and Blending are steps of this simplified stitching method.

Feature matching and image matching construct image registration procedure whose aim is to find one transformation matrix for each image. This matrix is used to fix the image in its proper position [41] and the blending step blends registered images especially in borders and removes seams [37], consequently. The steps of this simplified stitching method are explained in details in the following subsections.

#### 4.1.1. Feature Matching

At first, features must be extracted from image to match them at the next step. For feature extracting, Brown and Lowe [38] image stitching method uses SIFT [39] algorithm which contains following stages:

- *Scale-space extrema detection:* The difference-of-Gaussian function is used to search over all scales and image locations and identify potential interest points that are invariant to scale and orientation.
- *Key-point localization:* The potential interest points which are less stable and have low contrast or are poorly localized along an edge are rejected.
- *Orientation assignment:* In this stage, each point is assigned one or more orientation based on local image gradient directions. SIFT features are scale and orientation invariant because all future operations are performed on image data that has been transformed relative to its orientation, scale, and location.
- *Key-point descriptor:* To compute descriptor vectors for key-points such that the descriptors are highly distinctive and invariant to the remaining variations like shape distortion and illumination, the local image gradients are measured at the selected scale in the region around each key-point.

SIFT features are in a scale-space maxima/minima of a Difference of Gaussian (DOG) function. Each feature location has scale and orientation properties. Therefore, they have similarity-invariance in the frame which they are measured. The invariant descriptor is computed by accumulating local gradients in histograms of orientation. This causes the descriptor vector not to change with slight affine changes. This spatial accumulation is also important for shift invariance, since the interest point locations are typically only accurate in the 0–3 pixel range [42,43]. Using gradients that eliminates bias and normalizing the descriptor vector that eliminates gain achieve lead to illumination invariance. Since SIFT features are invariant under rotation and scale changes, this system can handle images with varying orientation and zoom.

#### 4.1.2. Image Matching

In this step, the goal is to find all matching images. The ones having more matching features than a constant number would be considered as potential match images of current image. First, the RANSAC method [44] is used to select a set of inliers that are compatible with a homography between the images. Next, a probabilistic model is applied to verify the match.

RANSAC is an iterative method to estimate parameters of a mathematical model from a set of data which contains outliers. In the case of image stitching, it selects sets of *4* feature correspondences and computes the homography *H* between them using the direct linear transformation (DLT) method [45]. This procedure repeats *n =* 500 times and the solution that has the maximum of inliers is selected. Given the probability that a feature match is correct between a pair of matching images (the inlier probability) is *p_i_*, the probability of finding the correct transformation after *n* trials is:

(1) $$p(H\;\mathrm{is}\;\mathrm{correct})=1-{\left(1-{\left(p_{i}\right)}^{r}\right)}^{n}$$

After a large number of trials the probability of finding the correct homography is very high. For example, for an inlier probability *p_i_* = 0.5, the probability that the correct homography is not found after 500 trials is approximately 1 × 10^−14^.

Brown and Lowe [38] use a probabilistic model for image matching verification which is explained in the following. For each pair of potentially matching images there are feature matches which are geometrically consistent (RANSAC inliers) and features that are inside the overlap area but not consistent (RANSAC outliers).

The idea of this verification model is to compare the probabilities of whether this set of inliers/outliers was generated by a correct image match or by a false image match. For a given image the total number of features in the area of overlap is denoted *n_f_* and the number of inliers is denoted *n_i_*. The probabilistic event that this image matches correctly/incorrectly is represented by the binary variable *m* ∈ {1,0}. The event that the *i^th^* feature match *f*^(*i*)^ ∈ {0,1} is an inlier/outlier is assumed to be independent Bernoulli so that the total number of inliers is binomial:

(2) $$p(f(1:n_{f})|m=1)=B(n_{i};n_{f},\;p_{1})$$

(3) $$p(f(1:n_{f})|m=0)=B(n_{i};n_{f},\;p_{0})$$

where *p_1_* is the probability of a feature being an inlier of a correct image match, and *p_0_* is the probability of a feature being an inlier of a false image match. The set of feature match variables {*f*(*i*),*i* = 1,2,…,*n_f_*} is denoted by *f*^(1:*n*_*f*_)^. The number of inliers is shown by $n_{i}=\sum_{i=1}^{n_{f}}f^{\left(i\right)}$ and *B(x; n*, *p)* is the Binomial distribution as follows:

(4) $$\begin{array}{l} B(x;n,\;p)=\frac{n!}{x!(n-x)!}p^{x}{\left(1-p\right)}^{n-x} \end{array}$$

Brown and Lowe [38] have chosen values *P_1_* = 0.6 and *P_0_* = *0.1.* Now the posterior probability that an image match is correct is evaluated using Bayes’ Rule:

(5) $$P(m=1|f^{\left(1:n_{f}\right)})=\frac{p(f^{\left(1:n_{f}\right)}|m=1)p(m=1)}{p(f^{\left(1:n_{f}\right)})}=1+\frac{p(f^{\left(1:n_{f}\right)}|m=0)p(m=0)}{p(f^{\left(1:n_{f}\right)}|m=1)p(m=1)}$$

An image match is accepted if *p*(*m* = 1|*f*^(1:*n*_*f*_)^) > *P*_min_

(6) $$\frac{B(n_{i};n_{f};p_{1})p(m=1)}{B(n_{i};n_{f};p_{0})p(m=0)}\;\begin{array}{c} \mathrm{accept}\\ >\\ <\\ \mathrm{reject} \end{array}\;\frac{1}{\frac{1}{p_{\text{min}}-1}}$$

Choosing values *p* (*m* = 1) = 10^−6^ and *p*_min_ = 0.999 gives the condition:

(7) $$n_{i}>\alpha +\beta \;n_{f}$$

For a correct image match, Brown and Lowe [38] have chosen *α = 8.0* and *β = 0.3*. At the end of this step, all low-resolution images are registered. It means that a transformation matrix is found for each image that transforms it to its correct position in the high-resolution-registered image. As the location of camera nodes are fixed, these matrixes could be used next time. Hence, the transformation matrix is sent to the related cluster head to give it the ability to register and finally stitch low-resolution images before sending them to Sink. It has two merits: first, after the first time calculation in Sink, there is no need to calculate these matrixes in cluster head and, second by stitching images before sending them, the network traffic between upper-tier and Sink is reduced significantly.

#### 4.1.3. Blending

The purpose of blending step is to make overlapped image edges disappear and it is necessary because even after a perfect image registration, that is practically impossible, some image edges are still visible due to some effects such as vignetting (intensity decreases towards the edge of the image), parallax effects due to unwanted motion of the optical center, radial distortion, etc.

Although there are good methods like the multi-band blending method presented by Brown and Lowe [38] which results in a very smooth and seamless image, the method used to blend the images in WMSNs should be simple. Whereas one of the stitching purposes in this application is to reduce the amount of transaction with Sink, stitching procedure should be performed in cluster heads which have resource limitations. In addition, it is not possible to send Brown and Lowe [38] multi-band maps to cluster heads because the stitching subset change by each query and new maps are needed. Besides, using different maps for each image in each query requires memory space in upper-tier nodes.

**Figure 4.**
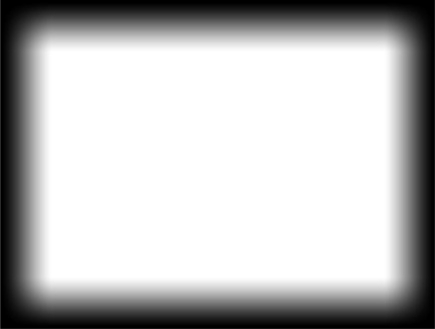
Predefined opacity map for blending.

Blending result is generated by overlapping images which have gradient transparency near the edges. So an opacity matrix is used for images as a map to determine opacity of pixels. Due to the fact that the nodes in lower-tier are homogeneous and the sizes of all images are equal, one predefined opacity map which is restored in upper-tier nodes is enough for all of the images. Figure 4 depicts this map.

Using a general map for all images has merits and demerits. As mentioned before, storing only one map in each cluster head memory is adequate to blend all the images. Also, it is possible to perform image stitching completely in cluster heads and avoid sending all images to Sink. Stitching images in cluster head reduces the size of data transmission and consequently decreases network traffic between upper-tier and Sink and prolongs nodes lifetime in upper-tier (see subsection 5.3). Nevertheless, the result of this method is not as uniform and smooth as the result image of other ones.

All in all, in the response to Sink queries, except the first one, cluster head gathers the low-resolution images of a proper subset of its camera nodes, applies the opacity map to them and transforms them by transformation matrices which have been sent to it after the first query by Sink. The result is a high-resolution image which is sent to Sink. How this proper subset of camera nodes is selected is described in the following.

### 4.2. Camera Sensor Node Selection Algorithm with Good Coverage and Minimum Energy Dissipation

As mentioned before, camera nodes in lower-tier are largely overlapped and a subset of them is adequate to create desired high-resolution image. Therefore, a new algorithm is proposed to be used in cluster head to provide a good coverage by selecting a subset of camera sensors. This algorithm is fair and creates a balance on energy consumption between lower-tier sensor nodes. It reduces the size of inter-tier transmission data and prolongs camera nodes lifetime in lower-tier. This algorithm uses a data structure called Coverage Data Structure whose creation procedure is explained in the following.

#### 4.2.1. Camera Node Selection Approach

As mentioned before, it is unnecessary to select all sensors of a cluster head to create a high resolution image and a proper subset of them is adequate. Selecting a proper subset of camera sensor nodes is solved by the presented algorithm. By each query from Sink, cluster head selects this subset based on some information (Coverage Data Structure) which has been received after first query in a way that in long term the average of activation times between camera sensor nodes becomes equal. Consequently, a balance in nodes energy consumption is achieved and this balance makes a good coverage of desired area be provided for a longer time. Other sensors that are not in this active subset are in sleep mode, so lower-tier energy dissipation decreases and its life time increases as much as possible. It is obvious that by reducing the number of active nodes to cover the desired area, inter-tier network traffic and camera overlap area are decreased. In addition, it reduces the processing amount in cluster head and response time to Sink queries.

**Figure 5.**
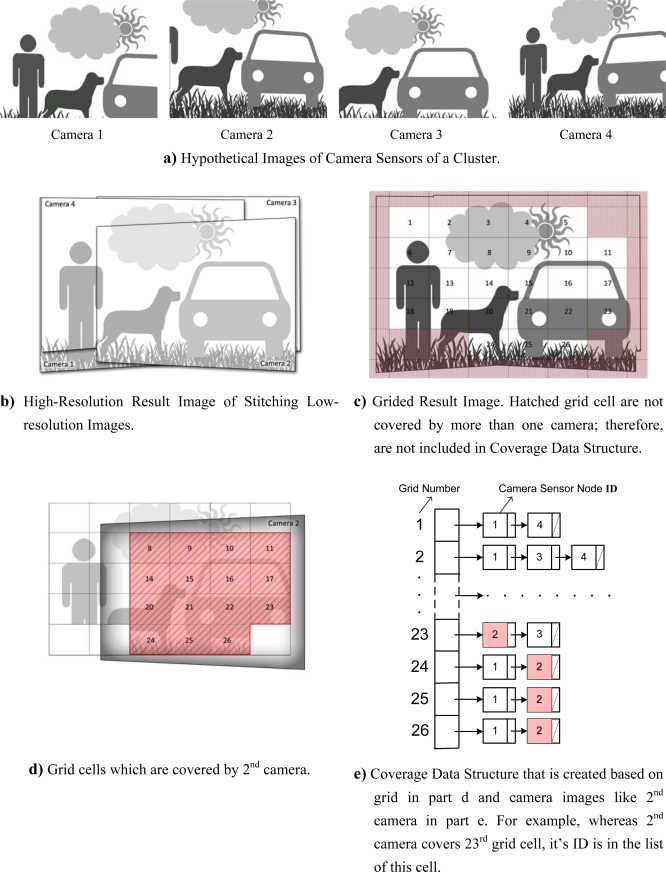
Creating coverage data structure.

Sink sends Coverage Data Structure to cluster heads along with stitching related information (transformation matrixes) in order to perform the presented algorithm. To calculate this information, after stitching low-resolution images captured by all camera sensors, Sink grids the final result that is a high resolution image (see Figure 5b). Also, it specifies which grid cells are covered by the image of each camera sensor (see Figure 5c,d). The result of this process is an array, which each element of it corresponds to a grid cell covered by more than one camera. Each element points to a linked list call Sensor List. Linked list (Sensor List) assigned to each grid cell (element) includes camera sensor nodes’ IDs which cover this gird cell. Figure 5e shows a sample of this data structure. Most of the grid cells are covered by multiple cameras because camera sensors have overlaps and each sensor image covers multiple gird cells. The grid cells which are not covered by more than one camera are not included in data structure. The reason is in case they were included, the corresponding cameras would be kept in active mode to cover them and the corresponding camera nodes would lose its energy quickly.

Also, the size of grid cells is an important issue. When a grid cell is not completely covered by a camera sensor node image, another camera must be selected to cover it. Consequently, the overlap of selected cameras expands. As the size of grid cells reduces more, the probability of covering them increase and less areas of camera images are wasted. By decreasing the size of grid cells, therefore, total overlap decreases and less number of camera sensor nodes is required to be in active mode to cover the desired area. However, decreasing the size of grid cells increases their number; consequently, the algorithm runtime prolongs and requires more memory.

If there are *N* grid cells, *M* camera nodes and in average each grid cell is covered by *K* camera nodes, in the best case, the algorithm finds a proper subset whose computational order is *O* (*N* × *K*). In the worst case, the proper subset is found which has a computational order of *O* (*M* × *N* × *K*). So, according to the memory volume and processing power of upper-tier nodes, the size of grid cells (*N*) should be specified. Also, *K* is achieved by Equation (8) after creating the Coverage Data Structure. In Equation (8), SensorList (i) is a linked list in Coverage Data Structure that includes all camera sensor nodes IDs which cover the i^th^ grid cell:

(8) $$K=\frac{1}{N}\times \sum_{i=1}^{N}\mathrm{length}\;\left(\mathrm{SensorList}(i)\right)$$

#### 4.2.2. Fair Camera Node Selection Algorithm

**Figure 6.**
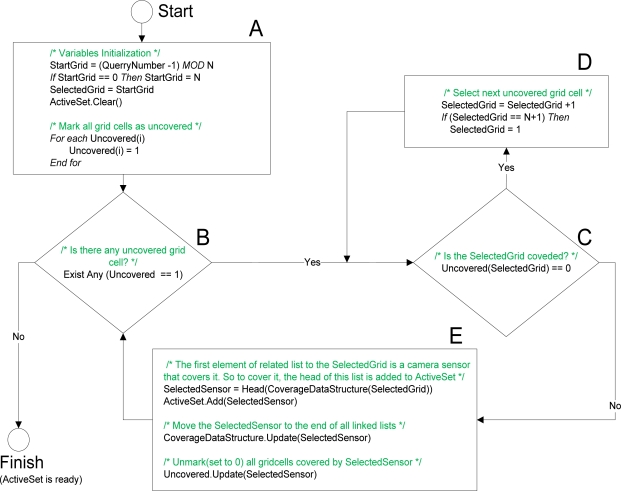
Flowchart of fire camera node selection algorithm.

After first query from Sink, and having all necessary information (transformation matrixes and Coverage Data Structure) which have been sent to the related cluster head, cluster head selects subset of camera sensor nodes based on the Fair Camera Node Selection algorithm to respond to subsequent queries. Presented algorithm selects this subset in a way that each node is selected almost equal times in multiple queries. It avoids the early failure of some sensor nodes and consequently a good coverage of field is provided for a longer time. The steps of Fair Camera Node Selection are explained in the following:

- *Initialization*: As mentioned before each camera sensor covers multiple grids, so after selecting a sensor to cover a specific grid cell, some other cells will be automatically covered by the same sensor and selecting another sensor to cover them is unnecessary. Accordingly, an array of flags called “***Uncovered Grids***” is needed to identify which grid cells are covered. Each element of this array corresponds to a grid cell and if an element in this array is marked (includes 1), it means that the corresponding grid cell is uncovered. If the element is unmarked (includes 0), it is covered by a camera in previous algorithm iterations. As is shown in Figure 6A, all elements of “***Uncovered Grids***” are marked (set to 1) at the beginning of the algorithm. In addition, “***Start Grid***” is a variable that identifies which grid cell must be covered first in this query and by each query it cycles between grid cells (see Figure 6A). This rotation between grid cells that changes the first grid cell to be covered causes a pseudo-random selection. Selected cameras are added to a set called “***Active Set***” which is empty at the beginning (see Figure 6A).
- *Checking the Answer*: If there is no marked grid cell in “***Uncovered Grids***” as an uncovered one, the algorithm task is completed and desired answer is in the “***Active Set***”. Otherwise, the grid cells which are uncovered must be covered in the next steps (see Figure 6B).
- *Finding an Uncovered Grid Cell*: Here, as there is still uncovered grid cell(s), we are sure that another camera must be selected. “***Selected Grid***” is a variable that identifies the grid which must be covered now and was set to “***Start Grid***” at the start of algorithm (see Figure 6A). If the “***Selected Grid***” is already covered (see Figure 6C), it must be set as the next uncovered grid (see Figure 6D) and this process goes on until an uncovered grid cell is found.
- *Camera Sensor Selection and Related Updates*: After the uncovered grid cell is found in previous step, in this step one of the cameras that cover it must be chosen. In the Coverage Data Structure, each grid cell has a list of sensor cameras covering it. First sensor node (header) in the sensor list of “***Selected Grid***” is selected as “***Selected Sensor***” and is added to the “***Active set***”. After this, as each camera sensor covers more than one grid cell, corresponding elements of all grid cells covered by “***Selected Sensor***” are unmarked (set to 0) in the “***Uncovered Grids***”. In addition, “***Selected Sensor***” is moved to the end of all sensor lists in the Coverage Data Structure in order to let the other sensors be selected in the next queries. To perform these tasks, a search through all lists is required (see Figure 6E). Then algorithm returns to *Checking the Answer* step.

At first it appears that using a random selection for uncovered grid cells instead of selecting them one after another (using “***Start Grid***” variable) is more fair but running tests have shown that they have no difference in results and all sensors are selected almost equal times. The result of this test is shown in subsection 5.2. Since random selection requires more processing and the result of both methods are almost the same, in order to decrease response time to Sink queries and preventing from processing power dissipation, it is better not to use it.

## 5. Analyses and Simulation Results

### 5.1. Simulation Environment

In order to simulate presented methods, OMNet++ [46] network simulator and MATLAB have been used. It is assumed that when the clustering algorithm is performed in upper-tier, all nodes are clustered in groups where most of them have *four* members. Also, a node is selected as a cluster head of each cluster such that the locations of cluster heads in entire upper-tier become uniform. It is supposed that each cluster head reach Sink after three hops. In order to create this hierarchical structure, HDA algorithm [28] is used. Whereas upper-tier sensor nodes are equipped with GPS, Sink knows their geographical position and sends the queries to the related cluster head.

The lower-tier consists of homogenous camera sensor nodes with image resolution 320 × 240 and it is assumed that each image is in gray-scale. As camera sensors in lower-tier are near to each other and are placed in almost same direction (see subsection 3.2), camera sensors of each cluster have considerable overlaps. It is assumed that each cluster in lower-tier has 24 members in average. Needless to say, sensor nodes in upper-tier are different from lower-tier nodes. Also, each camera node can reach its cluster head in upper-tier by one hop. In order to get precise results from the simulations, the parameters of CC2420 [47] Chipcon transceiver, listed in Table 1, are used for transceiver part of sensor nodes in both tiers.

**Figure 7.**
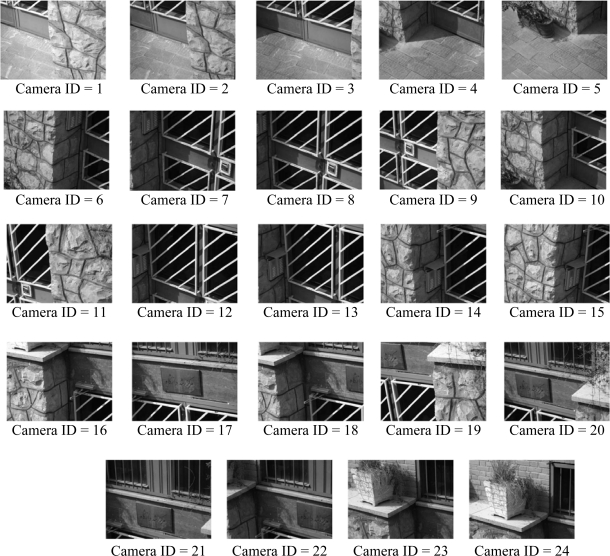
Assumed Camera Sensor Node Gray-Scale Images with Resolutions 320 × 240 Managed by Specific Cluster Head in Upper-Tier.

In the simulations, it is assumed that desired area is related to the specific cluster head. Initially, at first query from Sink, all low-resolution images of camera sensors in the related cluster head, which belong to requested area, are sent to Sink. Figure 7 shows the assumed low-resolution images which are captured from the desired area by camera sensor nodes that are connected to the specified cluster head. The images are captured from cameras which are placed in a 3-D field [with the size of around 3 m (width) × 3.5 m (height) × 1 m (depth)]. Besides, the distance of desired area (the door) and cameras in average is about 3.5 m.

**Figure 8.**
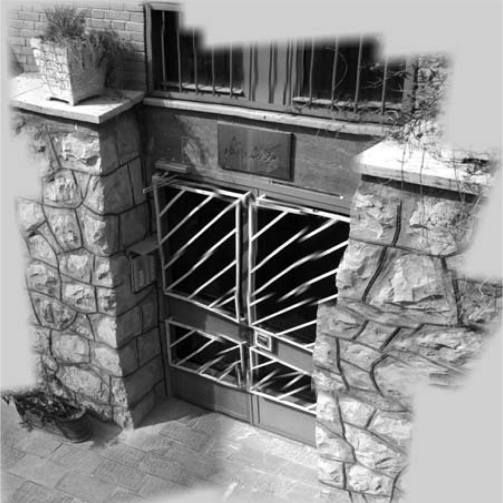
High-resolution and wide stitched image in sink.

After first query and stitching in Sink based on all low-resolution camera images that are related to specific cluster head, the results of stitching procedure are: transformation matrixes, A Coverage Data Structure and a high-resolution image. Using Equation (8) each grid cell in average covered by 3.68 cameras (*K* = 3.68). The high-resolution and wide stitched image produced from all low-resolution camera sensor images of desired area in Sink is shown in Figure 8.

As mentioned in subsection 4.2.1, Coverage Data Structure is created based on stitched high-resolution image gridded in Sink. In generating this data structure, the size of grid cells is assumed to be 80 × 60 so number of grid cells which are covered by more than one camera (*N*) is 288. In the following subsections, we are going to analyze several aspects of the proposed methods.

### 5.2. Analyses on the Fair Camera Node Selection (FCNS) Algorithm

**Figure 9.**
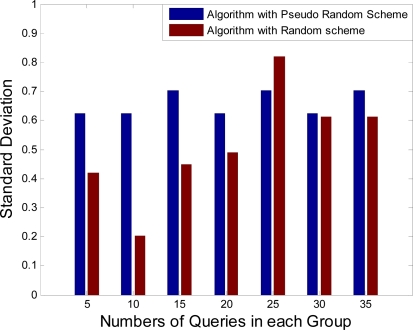
Computing standard deviation of camera activation times for each group of the queries.

In this subsection, performance and fairness of the presented node selection algorithm in subsection 4.2.2 is simulated and analyzed. Coverage Data Structure related to the high-resolution image in Figure 8 which is produced by stitching low-resolution images in Figure 7 are used in these simulations. At first, using a random selection for uncovered grid cells instead of selecting them sequentially is analyzed. It is assumed that Sink sends some queries to the specific cluster head and the standard deviation of camera sensor activation times are computed for each group of the queries. The computational results are shown in Figure 9. Using the random selection for uncovered grid cells or selecting them sequentially (pseudo-random) has no difference in results. Whereas computational overhead in pseudo-random selection is less than random selection, it is suitable for presented camera selection algorithm.

Simulation results in Figure 9 show that the presented algorithm has low standard deviation in camera sensor activation times (less than 1.0). Consequently, this algorithm is fair enough and selects camera sensor nodes in each group of queries almost equal times. However, to show that equalization of camera sensor activation times is useful in achieving a high-resolution image with complete coverage for a longer time, a greedy set cover algorithm [48] is compared with presented algorithm. The aim of greedy set cover algorithm is to cover entire desired area with minimum number of cameras which is named Minimum Camera Node Selection (MCNS). In this comparison, it is assumed that Sink sends multiple groups of queries to the specific cluster head and numbers of selected camera sensors are computed for each group of queries. The results of these computations are shown in Table 2.

Analyzing the results in Table 2 shows that for each group of the queries the average number of selected nodes by MCNS algorithm is lower than presented algorithm. But, often, MCNS algorithm selects sensor nodes that cover more grid cells than the other nodes. Therefore, some nodes are used more than others. Unbalanced usage of the camera nodes causes energy to diminish faster for nodes which are selected more. Consequently, by losing some nodes before the other ones, responding to queries with good coverage is impossible.

**Figure 10.**
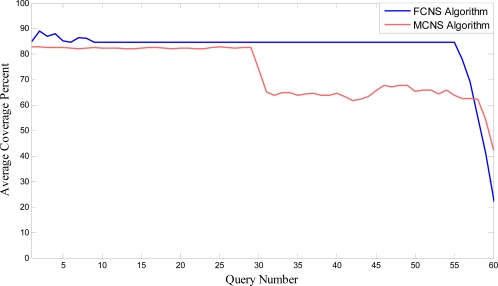
Comparing FCNS and MCNS algorithms.

To analyze the coverage issue in detail, another simulation is presented. The aim of this simulation is to compare coverage of desired area in many queries by presented algorithm and Minimum Camera Node Selection algorithm. The simulation has the same assumptions of subsection 5.1 (such as Coverage Data Structure, network topology) and it is related to a cluster head with 24 camera nodes. Also, it is assumed that all camera nodes have 2J energy at the beginning and according to Table 1 each camera will fail after 30 times of activation. Figure 10 depicts the average coverage percent of both algorithms in 60 queries.

As Figure 10 shows, the coverage percent of the presented algorithm is high in roughly the first 60 queries and then the coverage reduces extremely because many nodes fail almost together. But, coverage percent of MCNS reduces by about 20% after 30^th^ and 60^th^ queries because of some nodes failure. Simulation results show: although the presented algorithm gets minimum coverage more quickly, it provides good coverage for longer time in comparison with MCNS.

### 5.3. Analyzing Computational Overhead and Energy Efficiency in High Resolution Image Generation

In this subsection, the computational overhead of stitching low-resolution images, the energy efficiency of presented methods and quality of generated high resolution images are simulated and analyzed. It is assumed that after the first query and sending required information to the related cluster head, Sink sends 10 queries to cluster head and obtain high-resolution images in response to these queries. Also, as mentioned in subsection 5.1, related cluster head is located three hops far from Sink and transmission schemes in EQV-Architecture [49] are used to transmit stitched image to Sink through the upper-tier. As mentioned before, having the transformation matrices and blending maps, cluster head can stitch its camera sensor images with low computational overhead before sending them to Sink. In order to do so, every pixel of each selected low-resolution image must be transferred to its position in the high-resolution image by related transformation matrix and its opacity level in blending map be applied to them. Despite the result of simplified stitching method is not as good as the original one containing a multiband blending, it is worth utilizing because its low computational overhead lets the stitching be performed by cluster heads. For example, in this simulation, image stitching in cluster head by available transformation matrices requires approximately 21% computation of whole image stitching process from the beginning by comparing average run time of both algorithms in 10 run. On the other hand, reusing transformation matrices causes 79% computational performance.

**Figure 11.**
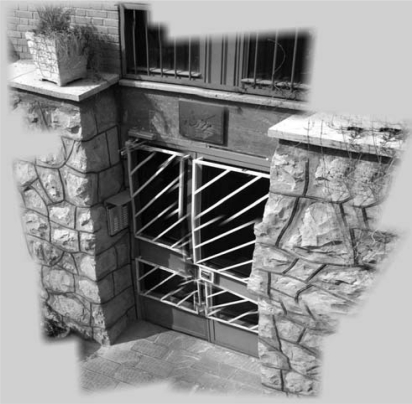
The stitched image in cluster head for the response to 8^th^ query.

Moreover, using the Coverage Data Structure, cluster head is able to select proper active subset of sensor nodes. Figure 11 shows the stitched image of 8^th^ query in the related cluster head. This high-resolution image is created using low-resolution images that generated with camera IDs 24, 21, 23, 19, 15, 17, 13, 8, 10, 9, 2, and 4.

As mentioned before, the grid cells covered by only one camera node are omitted from Coverage Data structure. Therefore, the stitched image in cluster head created by proper subset of camera sensor images has some small difference from stitched image created by all camera sensor images. However, based on simulation results shown in Figure 11, the stitched image in cluster head is acceptable for many monitoring sensor network applications.

**Figure 12.**
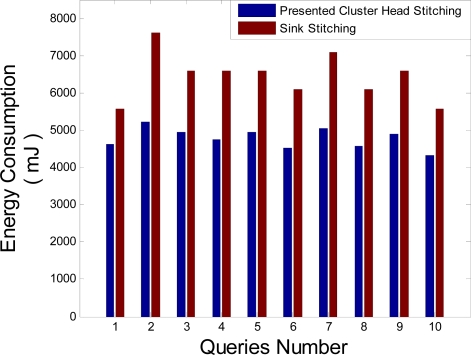
Network energy consumption for the queries 1 to 10.

Stitching in the cluster head presented in this literature omits transmitting redundant data to Sink and let save more energy in the network. To show this, it is assumed that 10 queries are sent to the desired area in the third hop far from Sink. In order to perform this simulation, energy consumption of each query is computed and compared with energy consumed in case stitching is done in Sink. Figure 12 which shows the simulation results of these computations says that the stitching in cluster head has less energy consumption compared to Sink stitching. Inserting these results into the Equation (9) shows that the optimization of the energy consumption using stitching in the cluster head is approximately 26%. In this equation, *Q* is the query numbers that arrived from Sink.

(9) $$\mathrm{EnergyEfficinecy}=\frac{\sum_{Q=1}^{10}{\left(\mathrm{Energy}\;\mathrm{Consumption}\;\mathrm{based}\;\mathrm{on}\;\mathrm{Sink}\;\mathrm{Stitching}\right)}_{Q}-\sum_{Q=1}^{10}{\left(\mathrm{Energy}\;\mathrm{Consumption}\;\mathrm{based}\;\mathrm{on}\;\mathrm{ClusterHead}\;\mathrm{Stitching}\right)}_{Q}}{\sum_{Q=1}^{10}{\left(\mathrm{Energy}\;\mathrm{Consumption}\;\mathrm{based}\;\mathrm{on}\;\mathrm{Sink}\;\mathrm{Stitching}\right)}_{Q}}$$

## 6. Conclusions

In this article, a high-resolution and wide image of desired area based on the proposed methods and a new designed two-tier network topology structure has been presented. Also, energy consumption that is very important issue in WMSNs has been considered. The presented two-tier network topology has utilized sensor nodes with low-resolution camera only in lower-tier and stronger sensor nodes without camera in upper-tier. The proposed network topology structure causes energy dissipation to decrease as much as possible.

The presented camera sensor selection algorithm causes equal energy consumption between camera nodes because it selects camera sensor nodes in a fair manner to produce an active camera subset. Consequently, the high-resolution and wide image with complete coverage of desired area is obtained for a longer time. Also, the proposed stitching method in the cluster head stitches the low-resolution camera sensor images and sends only a high resolution stitched image to Sink without redundant information. The simulation results show that stitching low-resolution images in the cluster head leads to 26% of energy efficiency. All in all, in this paper, using image stitching in WMSNs led to minimization of the energy dissipation and the obtained high-resolution image is suitable to satisfy many monitoring camera-based WMSNs applications.

## Figures and Tables

**Table 1.** CC2420 Transceiver parameters.

**Parameter**	**Value**
Bit Rate	250 k_bps_
Listen Power	60 mJ/sec
Receive Power	63 mJ/sec
Transmission Power	57 mJ/sec
Setup Time	1msec
Communication Distance	300m

**Table 2.** Numbers of camera sensor selections.

**Query Number = 15**	**Camera ID**	**1**	**2**	**3**	**4**	**5**	**6**	**7**	**8**	**9**	**10**	**11**	**12**
	Camera Selection Times with FCNS Algorithm	8	7	8	7	8	8	9	8	7	7	8	8
	Camera Selection Times with MCNS Algorithm	8	7	8	7	8	0	0	15	7	15	0	6
**Query Number = 15**	**Camera ID**	**13**	**14**	**15**	**16**	**17**	**18**	**19**	**20**	**21**	**22**	**23**	**24**
	Camera Selection Times with FCNS Algorithm	7	8	8	8	6	8	8	8	8	8	9	9
	Camera Selection Times with MCNS Algorithm	6	3	15	0	0	0	15	15	11	4	15	0
**Query Number = 30**	**Camera ID**	**1**	**2**	**3**	**4**	**5**	**6**	**7**	**8**	**9**	**10**	**11**	**12**
	Camera Selection Times with FCNS Algorithm	15	15	15	15	15	15	16	16	15	15	15	15
	Camera Selection Times with MCNS Algorithm	15	15	14	16	12	0	0	30	18	30	0	9
**Query Number = 30**	**Camera ID**	**13**	**14**	**15**	**16**	**17**	**18**	**19**	**20**	**21**	**22**	**23**	**24**
	Camera Selection Times with FCNS Algorithm	15	15	16	15	14	15	16	15	16	15	17	16
	Camera Selection Times with MCNS Algorithm	10	11	30	0	0	0	30	30	14	16	30	0
